# The uneven playing field: provider participation and regional disparities in oral health examination rates in Korea

**DOI:** 10.4178/epih.e2025012

**Published:** 2025-03-10

**Authors:** Hye-Lim Hong, Nam-Hee Kim

**Affiliations:** 1 Department of Dental Hygiene, Yonsei University Graduate School, Wonju, Korea; 2 Department of Dental Hygiene, Yonsei University Mirae Campus, Wonju, Korea

**Keywords:** Community health, Delivery of health care, Oral health, Regional disparities

## Abstract

**OBJECTIVES:**

This study investigated regional disparities in adult oral health examination rates in Korea, despite free oral health screenings by the National Health Insurance Service (NHIS). It focused on the impact of provider factors, such as the availability of dental clinics and non-dental institutions.

**METHODS:**

A cross-sectional analysis of 2022 data from 229 districts was conducted. The dependent variable was the adult oral health examination rate, while independent variables included provider factors, community health status, lifestyle, demographic, and socioeconomic characteristics. Descriptive statistics, Pearson’s correlation, and multiple regression analyses identified significant predictors.

**RESULTS:**

Non-metropolitan areas had higher oral health examination rates (27.4%) than metropolitan areas (25.3%). Correlation analysis showed the general health examination rate (r=0.583) and the number of screening institutions (r=0.234) were the strongest predictors (p<0.001). Regression analysis showed a 1% increase in general health examination rates led to a 1.44% rise in oral health examination rates (p<0.001).

**CONCLUSIONS:**

Despite NHIS policies, significant regional disparities persist, showing that providing screenings alone is insufficient. Integrating oral health screenings with general health examinations is necessary. Policymakers must promote collaboration between dental and non-dental providers to ensure equitable, integrated health services, enhancing preventive care and reducing disparities.

## GRAPHICAL ABSTRACT

[Fig f3-epih-47-e2025012]

## Key Message

This study analyzed adult oral health examination rates across 229 districts in Korea and confirmed the existence of substantial regional disparities. Integrating oral health screenings into general health check-ups and expanding the participation of non-dental institutions were proposed as effective strategies for improving regional accessibility and equity.

## INTRODUCTION

Oral health is a crucial component of overall health, significantly affecting individuals’ quality of life and well-being. Despite its importance, oral health often receives less attention in public health policies than general health, resulting in disparities in access to and utilization of preventive services [1,2]. To address this issue, Korea implemented the National Health Screening Program in 1995, aimed at promoting public health and enabling early detection of diseases [3]. Recognizing the need for comprehensive oral health strategies, oral health screening was incorporated into the national program in 2002 to identify and prevent oral diseases, including caries and periodontal diseases, at an early stage [4].

Adult oral health screenings are provided biennially, or annually for non-office workers, and are entirely funded by the National Health Insurance Service (NHIS) since 2008, with additional financial support from local governments for Medical Aid recipients [5]. Despite the elimination of economic barriers and increased accessibility due to policy reforms, participation in adult oral health examinations has paradoxically decreased—from 31.7% in 2017 to 26.5% in 2022. This decline is in stark contrast to the general health screening rate, which has consistently remained above 75% during the same period [6].

The disparity in screening rates between general and oral health services can largely be attributed to the voluntary nature of oral health examinations, in contrast to the mandatory general health screenings required under the Industrial Safety and Health Act [7]. This discrepancy raises concerns about the effectiveness of existing oral health policies and suggests that individuals perceive less value or necessity in oral health screenings when not mandated. This phenomenon is further compounded by differences in individual factors [8-10], such as age, education, previous dental experiences, and regional factors, notably the availability and distribution of dental care providers [11]. Additionally, regional differences independently affect the dental care utilization of Americans aged 50 and above, regardless of their socioeconomic status [12]. These factors significantly influence health behaviors and service utilization, determining whether individuals seek preventive oral care [13,14].

Healthcare utilization is influenced by a variety of factors including gender, age, ethnicity, and geographical accessibility [11]. Previous research has identified three critical components necessary for improving access to dental care: patient demand, provider-related factors, and economic factors, which encompass financial support and insurance coverage. Among these, provider-related factors are particularly significant. While the focus is often on the number of dentists, other metrics such as the number of institutions and their service participation rates are equally important. These provider-related factors have been widely acknowledged in previous studies as key determinants of healthcare utilization, underscoring their role in facilitating access to preventive oral care services [15].

Existing literature has extensively explored individual determinants of oral health behaviors; however, relatively few national studies have focused on regional provider factors and their role in mediating disparities in oral health examination rates [16-18]. This study sought to address this gap by systematically examining the impact of regional provider factors—including the number of dental clinics, dentists, and the availability of oral health institutions—on adult oral health examination participation across 229 districts in Korea. A distinctive aspect of this study is that it highlights the complex interplay between provider factors and other contextual variables, thereby offering a novel contribution to the understanding of structural determinants of oral health disparities.

By focusing on provider-related determinants, this research not only identifies key factors influencing oral health examination rates but also proposes targeted policy interventions to bridge the existing regional disparities. The findings are expected to inform the development of more effective, region-specific strategies to promote equitable access to oral health services and ultimately contribute to the improvement of national oral health outcomes.

## MATERIALS AND METHODS

### Study design and subjects

This study utilized a cross-sectional design and secondary data to explore regional disparities in adult oral health examination rates throughout Korea. The analysis encompassed 229 administrative districts, including both metropolitan and non-metropolitan areas. Data regarding adult oral health examination rates for the year 2022 were sourced from the NHIS database [6]. Additional regional-level data, such as demographic and socioeconomic characteristics, were sourced from the Korean Statistical Information Service [19]. To further validate the findings and enhance the robustness of the analysis, a supplemental telephone survey was conducted, targeting healthcare institutions in regions with low participation rates.

### Variables and operational definitions

The primary dependent variable in this study was the adult oral health examination rate, which is defined as the proportion of adults who received oral health examinations out of those eligible for screening in each district. To enable comparisons across regions with different population sizes, this measure was standardized.

Independent variables were categorized into five domains based on a conceptual framework adapted from Lalonde’s model of health determinants [20] and Dever’s community health model [21]. These domains are described below (Table 1):

• Oral health care system (provider factors): (1) Number of dentists per 100,000 population [22]; (2) Number of oral health examination institutions per 100,000 population [23]; (3) Proportion of oral health examinations performed at dental institutions [22]; (4) Proportion of oral health examinations performed at non-dental institutions (e.g., hospitals, general hospitals, public health centers) [22]

• Community health status [24]: (1) Chewing discomfort rate; (2) Depression experience rate; (3) Subjective health perception rate

• Lifestyle factors: (1) Smoking rate; (2) Monthly drinking rate; (3) Walking practice rate; (4) Brushing after lunch practice rate; (5) General health examination rate

• Demographic characteristics: (1) Average age [25]; (2) Population density [26]

• Socioeconomic characteristics: (1) Employment rate [27]; (2) Financial independence rate [28]

### Statistical analysis

The spatial distribution of adult oral health examination rates was visualized using Geographic Information System version 3.36.3 (R Foundation for Statistical Computing, Vienna, Austria). Descriptive statistics were calculated to summarize the distribution of oral health examination rates and key independent variables across the 229 districts. Pearson correlation coefficients were used to explore the relationships between oral health examination rates and the selected regional variables.

To identify the factors significantly influencing oral health examination rates, multiple regression analysis was conducted using a hierarchical approach. In model I, only provider factors were included. Models II, III, IV, and V sequentially added community health status, lifestyle, demographic, and socioeconomic variables to the provider factors. Finally, model VI included all variables to conduct a comprehensive assessment of the determinants of adult oral health examination rates. Multicollinearity was tested using the variance inflation factor (VIF), and only variables with a VIF<5 were retained to ensure the stability of the regression models [29].

All statistical analyses were performed using R version 4.3.3 (R Foundation for Statistical Computing, Vienna, Austria), and a two-tailed p-value<0.05 was considered statistically significant for all tests.

### Ethics statement

This study utilized secondary data from publicly accessible national databases; hence, approval from the Institutional Review Board was not required. Nonetheless, the study complied with ethical guidelines for using secondary data in research, maintaining data anonymity and confidentiality during the analysis [30].

## RESULTS

### Descriptive analysis of oral health examination rates and provider factors

**Figure 1.**
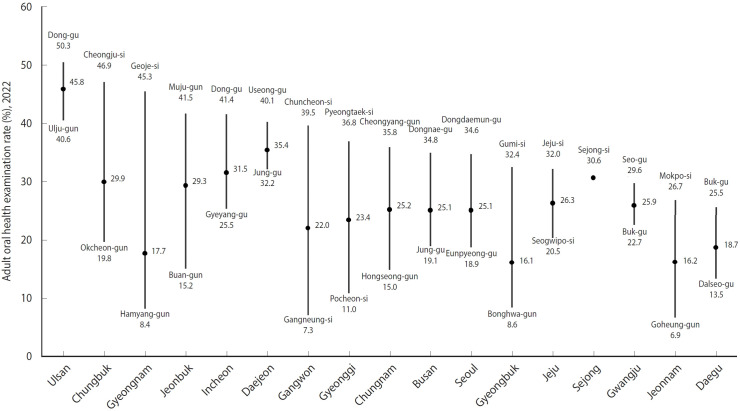
Regional disparities in the utilization rates of oral health examinations across 17 provinces.

**Figure 2.**
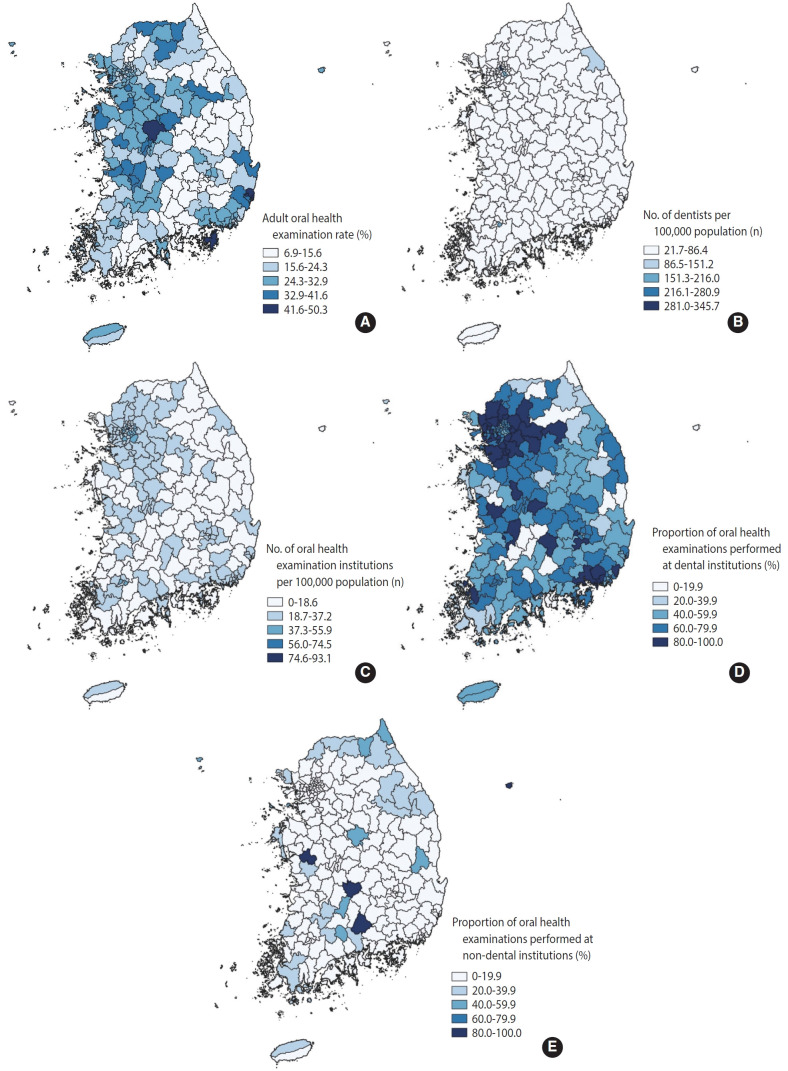
Geographical distribution of oral health examination and oral health care system variables. (A) Adult oral health examination rate. (B) Number of dentists per 100,000 population. (C) Number of oral health examination institutions per 100,000 population. (D) Proportion of oral health examinations performed at dental institutions. (E) Proportion of oral health examinations performed at non-dental institutions.

The adult oral health examination rate varied significantly across the 229 districts included in this study, highlighting notable regional disparities (Figures 1 and 2, Table 2). The average screening rate was 23.56±8.99%, with rates ranging from a minimum of 6.86% to a maximum of 50.28%. This indicates a relative disparity of 7.33-fold between the districts with the lowest and highest rates. Provincially, Ulsan recorded the highest mean examination rate at 45.2%, while Daegu had the lowest at 18.9%, reflecting a substantial absolute provincial disparity of 26.3 percentage points. Additionally, non-metropolitan areas consistently exhibited higher screening rates compared to metropolitan regions such as Seoul, Incheon, and Gyeonggi. Specifically, non-metropolitan districts reported an average rate of 27.4%, in contrast to 25.3% in metropolitan districts.

In terms of provider-related factors, the average number of dental institutions conducting oral health examinations was 21.70±11.17 per 100,000 population. However, this figure varied significantly across different regions, ranging from zero institutions in Yeongdeok-gun, Gyeongsangbuk-do, to 93.1 institutions in Jung-gu, Seoul. Similarly, the number of dentists per 100,000 population showed considerable variation, with an average of 50.33±35.07 dentists across districts. This ranged from 21.67 in Yangyang-gun, Gangwon-do, to 345.74 in Jung-gu, Daegu.

The proportion of oral health examinations performed at dental institutions, a measure of service availability, was 60.51±21.73%. In Hoengseong-gun, Gangwon-do, all dental institutions offered oral health screenings, whereas in 10 districts, including Cheongyang-gun and Namhae-gun, no dental institutions provided such services. In contrast, the proportion for non-dental healthcare institutions was significantly lower, averaging only 10.57±16.65%. Notably, in four districts—Cheongyang-gun, Ulleung-gun, Sancheong-gun, and Muju-gun—all non-dental institutions conducted oral health examinations, compensating for the lack of dental providers.

### Correlation analysis between oral health examination rates and regional variables

The Pearson correlation analysis identified multiple significant correlations between the rates of adult oral health examinations and the selected independent variables (Table 3). Notably, the rate of general health examinations exhibited the strongest positive correlation (r=0.583, p<0.001), indicating that individuals who undergo general health examinations are also more inclined to participate in oral health screenings.

In addition, positive correlations were observed with the number of dentists (r=0.120, p=0.069) and oral health examination institutions (r=0.234, p<0.001), indicating that regions with more service providers had higher screening rates.

Negative correlations were identified with average age (r=-0.496) and employment rate (r=-0.251), indicating that older adults and individuals in highly employed regions were less likely to participate in oral health screenings (p<0.001).

### Multiple regression analysis of factors affecting oral health examination rates

Multiple regression analysis was conducted to identify the independent factors that significantly influence adult oral health examination rates across 229 districts (Table 4). In model I, which included only provider-related factors, the proportion of oral health examinations performed at non-dental institutions emerged as a significant predictor (coefficient=0.159, p<0.001). However, the inclusion of lifestyle and demographic variables in models III and IV, respectively, diminished the impact of provider factors. This shift increased the explanatory power of the models, underscoring the importance of broader contextual factors.

In the final model (model VI), which included all variables, two key determinants were found to significantly impact adult oral health examination rates: the general health examination rate (coefficient=1.436, p<0.001) and the proportion of oral health examinations performed at non-dental healthcare institutions (coefficient=0.147, p<0.001). This positive correlation between general health examination rates and oral health examination participation highlights the potential advantages of integrated screening programs.

## DISCUSSION

This study examined regional disparities in adult oral health examination rates across 229 districts in Korea, with a focus on the impact of provider factors such as the number of dental institutions, dentists, and the participation rate of non-dental institutions that offer oral health screenings. The findings highlighted significant disparities between metropolitan and non-metropolitan regions, indicating structural barriers that hinder equitable access to preventive oral healthcare, even though the NHIS provides these services at no cost.

In this study, we observed a strong correlation between the rates of adult oral health examinations and general health examinations. This result aligns with prior research that underscores the synergistic benefits of integrated screening programs [31-34]. Additionally, the association of higher average age and employment rate with lower oral health examination rates indicates a need for targeted interventions aimed at older adults [35,36] and workers [8].

Based on the results of multiple regression analysis and a supplemental telephone survey, regions without dedicated dental institutions but with non-dental institutions providing oral health screenings reported higher participation rates. This suggests that involving non-dental healthcare institutions in oral health screening could be an effective strategy to improve access in underserved areas. However, several barriers may impede the participation of non-dental institutions in these screenings. These barriers include inadequate training for conducting screenings, a lack of necessary dental equipment, and insufficient financial or policy incentives to encourage participation. Addressing these challenges through targeted training programs, investments in infrastructure, and the development of incentive structures could further enhance the role of non-dental institutions in reducing health disparities.

One of the key issues highlighted is that not all dental clinics are actively participating in the NHIS oral health screening program, despite being eligible. This situation mirrors challenges seen in the United States Medicare system, where although Medicaid coverage has expanded, it is primarily in areas that already have a higher concentration of dental providers [37,38]. This expansion further exacerbates disparities in regions with fewer dental providers or lower accessibility. Moreover, a significant number of dental providers do not accept Medicaid patients, which contributes to similar access issues [39,40]. The reasons for low participation may include inadequate reimbursement rates, high administrative burdens, and the voluntary nature of enrollment, factors that collectively undermine the NHIS’s effectiveness in ensuring comprehensive coverage [41]. This limited engagement among providers worsens regional disparities and diminishes the likelihood that individuals in underserved areas will receive preventive care.

Moreover, this study found that integrating oral health services into general health screenings and broadening the role of non-dental institutions significantly improved oral health examination rates. These findings align with those of a previous study [31], which highlighted the benefits of integrating oral health management into primary healthcare systems to enhance service accessibility. Insights from general health screenings underscore the advantages of mandatory participation policies, which have successfully ensured high levels of accessibility and engagement. Adopting similar policies for oral health screenings could help mitigate disparities. While general health screenings benefit from mandatory participation and streamlined processes, oral health screenings are voluntary and lack comparable policy support, resulting in lower participation rates. This discrepancy highlights the urgent need for structural reforms to better integrate oral health screenings with general health programs. The strong correlation between the involvement of non-dental healthcare institutions and higher screening rates suggests that these institutions can effectively bridge the service gap. This observation indicates that strategies focusing solely on increasing the number of dental providers might be inadequate. Instead, a more comprehensive approach that utilizes existing healthcare infrastructure, such as public health centers, and encourages non-dental providers to engage in oral health screenings could be an effective strategy to reduce disparities [32].

Policy implications necessitate addressing the regulatory framework that governs provider participation in the NHIS oral health screening program [1]. Current policies should be revised to foster more consistent provider engagement by increasing reimbursement rates and simplifying administrative processes. Mandating participation, similar to what is required in general health screenings, could ensure equitable service availability, especially in underserved areas. Implementing these measures would help to reduce disparities and ensure that all eligible adults have access to oral health screenings, regardless of their place of residence [33].

Strengthening collaboration between dental and non-dental providers is crucial for ensuring equitable access to integrated health services. The strong correlation between general and oral health examination rates suggests that integrated screening programs can effectively promote participation in both types of care [42]. These findings contribute to the broader public health discourse by addressing structural barriers and proposing actionable strategies to reduce regional disparities. Policymakers should therefore consider models that facilitate combined health services, which could increase efficiency and reduce the overall burden on individuals seeking preventive care [34].

In conclusion, this study highlights substantial regional disparities in the rates of adult oral health examinations across Korea, despite the NHIS offering free access to these services. The findings highlight that simply having a policy in place does not ensure equitable access, as disparities are primarily fueled by the uneven distribution and participation of providers. Although metropolitan areas typically boast a higher density of dental clinics, this advantage does not correspond to increased screening rates, suggesting that access is affected not only by availability but also by provider engagement.

The results suggest that integrating oral health screenings into general health services and expanding the role of non-dental institutions can effectively address disparities in underserved areas. Policy interventions encouraging broader provider participation, mandating service provision, and integrating oral health into comprehensive screening programs are essential for improving accessibility and promoting oral health equity nationwide. By addressing these structural issues, Korea can make significant strides toward ensuring that all adults, regardless of region, have equal access to preventive oral healthcare.

### Limitations and future research directions

This study has several limitations. First, its cross-sectional design prevents the establishment of causal relationships between provider factors and oral health examination rates. Longitudinal studies are necessary to evaluate how changes in provider participation and regional health policies affect oral health outcomes over time. Second, while the study included multiple contextual variables, it did not account for potentially influential unmeasured factors such as individual health beliefs, oral health literacy, and cultural norms. Future research should investigate these qualitative dimensions to offer a more detailed understanding of the barriers to participation in oral health screenings [32].

## Figures and Tables

**Figure f3-epih-47-e2025012:**
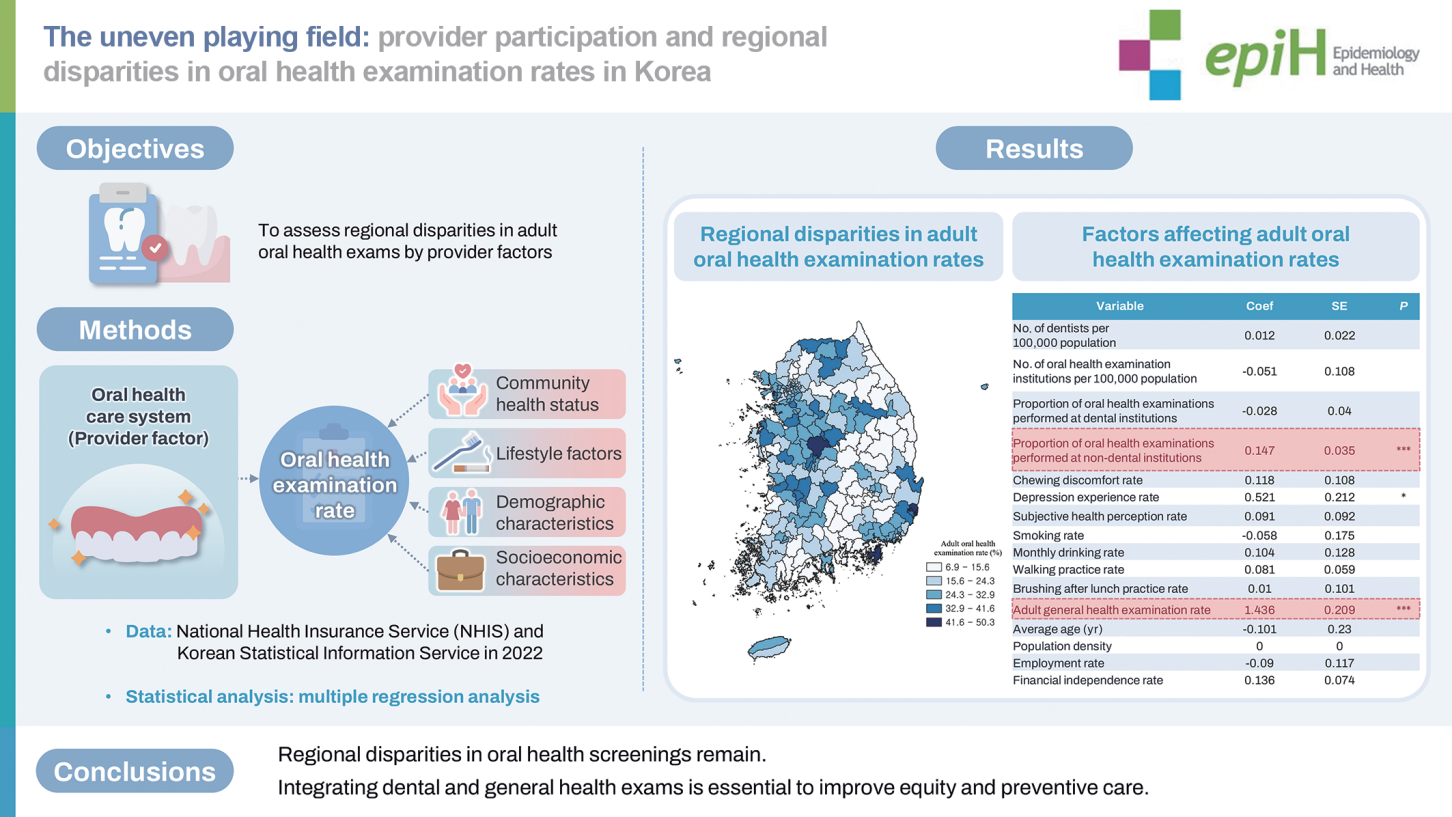


**Table 1.** The variables in the study

Variables	Definitions
Dependent	
Adult oral health examination rate	Proportion of people utilizing oral health examinations among those eligible
Independent	
Oral health care system (provider factors)	
No. of dentists per 100,000 population	(No. of dentists in the region/Regional population)×100,000
No. of oral health examination institutions per 100,000 population	(No. of oral health screening institutions in the region/Regional population)×100,000
Proportion of oral health examinations performed at dental institutions	(No. of dental facilities that provide oral health examinations/Total no. of dental facilities in the region)×100
Proportion of oral health examinations performed at non-dental institutions	(No. of non-dental facilities that provide oral health examinations/Total no. of non-dental facilities in the region)×100
Community health status	
Chewing discomfort rate	Proportion of people aged 65 and older who perceive difficulty in chewing due to problems
Depression experience rate	Proportion of people who experienced depressive symptoms for two or more weeks in the past
Subjective health perception rate	Proportion of people who reported their health as “good” or “very good”
Lifestyle factors	
Smoking rate	Proportion of people who have smoked 100 cigarettes or more in lifetime and currently smoke
Monthly drinking rate	Proportion of people who drank alcohol at least once a month in the past year
Walking practice rate	Proportion of people who walked for at least 30 min/day, 5 or more days in the past week
Brushing after lunch practice rate	Proportion of people who brushed their teeth after lunch yesterday
Adult general health examination rate	Proportion of people utilizing health examinations among those eligible
Demographic characteristics	
Average age	Average age of the total population in the region
Population density	Population per unit area in the regions
Socioeconomic characteristics	
Employment rate	Proportion of employer aged 15 and older
Financial independence rate	Proportion of a local government’s budget that is funded by its own sources

**Table 2.** Descriptive statistics of variables associated with adult oral health examinations

Variables	Mean±SD	Min	Max	EQ
Adult oral health examination rate	23.56±8.99	6.86	50.28	7.33
No. of dentists per 100,000 population	50.33±35.07	21.67	345.74	15.95
No. of oral health examination institutions per 100,000 population	21.70±11.17	0	93.10	0
Proportion of oral health examinations performed at dental institutions	60.51±21.73	0	100	0
Proportion of oral health examinations performed at non-dental institutions	10.57±16.65	0	100	0
Chewing discomfort rate	29.97±5.46	11.90	44.40	3.73
Depression experience rate	7.24±2.32	1.70	13.50	7.94
Subjective health perception rate	48.58±5.86	37.20	69.20	1.86
Smoking rate	20.39±3.19	11.20	30.70	2.74
Monthly drinking rate	57.46±4.53	41.00	66.50	1.62
Walking practice rate	47.60±11.07	22.20	72.30	3.26
Brushing after lunch practice rate	67.83±5.06	54.40	83.10	1.53
Adult general health examination rate	73.96±3.35	63.50	82.40	1.30
Average age (yr)	47.33±4.96	37.70	58.70	1.56
Population density	3,711.45±5,813.97	19.11	25,007.79	1,308.62
Employment rate	64.01±6.27	49.30	82.40	1.67
Financial independence rate	19.76±11.88	6.20	61.10	9.85

SD, standard deviation; Min, minimum; Max, maximum; EQ, extremal quotient.

**Table 3.** Correlations between variables and the adult oral health examination rate

Variables	Adult oral health examination rate	
	r	p-value
No. of dentists per 100,000 population	0.120	0.069
No. of oral health examination institutions per 100,000 population	0.234	<0.001
Proportion of oral health examinations performed at dental institutions	0.167	0.011
Proportion of oral health examinations performed at non-dental institutions	0.097	0.142
Chewing discomfort rate	-0.138	0.036
Depression experience rate	0.233	<0.001
Subjective health perception rate	-0.026	0.690
Smoking rate	-0.162	0.014
Monthly drinking rate	0.302	<0.001
Walking practice rate	0.266	<0.001
Brushing after lunch practice rate	0.183	0.005
Adult general health examination rate	0.583	<0.001
Average age (yr)	-0.496	<0.001
Population density	0.169	0.010
Employment rate	-0.251	<0.001
Financial independence rate	0.340	<0.001

**Table 4.** Multiple regression of variables and the adult oral health examination rate^1^

Variables	Model I	Model II	Model III	Model IV	Model V	Model VI
No. of dentists per 100,000 population	0.000 (0.027)	0.005 (0.027)	0.024 (0.022)	0.003 (0.024)	-0.004 (0.026)	0.012 (0.022)
No. of oral health examination institutions per 100,000 population	0.166 (0.103)	0.121 (0.106)	-0.010 (0.096)	-0.054 (0.110)	-0.063 (0.110)	-0.051 (0.108)
Proportion of oral health examinations performed at dental institutions	0.090 (0.045)^*^	0.081 (0.044)	-0.002 (0.039)	-0.012 (0.043)	0.048 (0.044)	-0.028 (0.040)
Proportion of oral health examinations performed at non-dental institutions	0.159 (0.042)^***^	0.164 (0.041)^***^	0.138 (0.034)^***^	0.143 (0.037)^***^	0.175 (0.041)^***^	0.147 (0.035)^***^
Chewing discomfort rate		-0.153 (0.119)				0.118 (0.108)
Depression experience rate		0.807 (0.252)^**^				0.521 (0.212)^*^
Subjective health perception rate		-0.052 (0.107)				0.091 (0.092)
Smoking rate			-0.080 (0.161)			-0.058 (0.175)
Monthly drinking rate			0.167 (0.119)			0.104 (0.128)
Walking practice rate			0.111 (0.051)^*^			0.081 (0.059)
Brushing after lunch practice rate			0.029 (0.099)			0.010 (0.101)
Adult general health examination rate			1.568 (0.164)^***^			1.436 (0.209)^***^
Average age (yr)				-1.120 (0.136)^***^		-0.101 (0.230)
Population density				0.000 (0.000)		-0.000 (0.000)
Employment rate					-0.351 (0.117)^**^	-0.090 (0.117)
Financial independence rate					0.251 (0.062)^***^	0.136 (0.074)
R^2^	0.114^***^	0.163^***^	0.441^***^	0.322^***^	0.202^***^	0.480^***^
Adjusted R^2^	0.098	0.136	0.418	0.303	0.180	0.440

Values are presented as coefficient (standard error).

1 Model I: A model consisting of oral health care system variables; Model II: A model consisting of oral health care system and community health status variables; Model III: A model consisting of oral health care system and lifestyle variables; Model IV: A model consisting of oral health care system and demographic variables; Model V: A model consisting of oral health care system and socioeconomic variables; Model VI: A model consisting of all variables: oral health care system, community health status, lifestyle, demographic, and socioeconomic variables.

* p<0.05,

** p<0.01,

*** p<0.001.
